# Variable Structure Controller for Energy Savings in an Underwater Sensor Platform

**DOI:** 10.3390/s24175771

**Published:** 2024-09-05

**Authors:** João Falcão Carneiro, João Bravo Pinto, Fernando Gomes de Almeida, Nuno A. Cruz

**Affiliations:** 1 Instituto de Ciência e Inovação em Engenharia Mecânica e Engenharia Industrial, Faculdade de Engenharia, Universidade do Porto, Rua Dr. Roberto Frias, s/n, 4200-465 Porto, Portugal; fga@fe.up.pt; 2 Faculdade de Engenharia, Universidade do Porto, Rua Dr. Roberto Frias, 400, 4200-465 Porto, Portugal; jpp.professional@gmail.com (J.B.P.); nacruz@fe.up.pt (N.A.C.); 3 Instituto de Engenharia de Sistemas e Computadores, Tecnologia e Ciência, Faculdade de Engenharia, Universidade do Porto, Rua Dr. Roberto Frias, 4200-465 Porto, Portugal

**Keywords:** variable structure controllers, autonomous underwater vehicles, variable buoyancy, energy savings, depth control

## Abstract

This paper introduces a new variable structure controller designed for depth control of an autonomous underwater sensor platform equipped with a variable buoyancy module. To that end, the prototype linear model is presented, and a finite element-based method is used to estimate one of its parameters, the hull deformation due to pressure. To manage potential internal disturbances like hull deformation or external disturbances like weight changes, a disturbance observer is developed. An analysis of the observer steady-state estimation error in relation to input disturbances and system parameter uncertainties is developed. The locations of the observer poles according to its parameters are also identified. The variable structure controller is developed, keeping energy savings in mind. The proposed controller engages when system dynamics are unfavorable, causing the vehicle to deviate from the desired reference, and disengages when dynamics are favorable, guiding the vehicle toward the target reference. A detailed analysis determines the necessary switching control actions to ensure the system reaches the desired reference. Finally, simulations are run to compare the proposed controller’s performance with that of PID-based controllers recently developed in the literature, assessing dynamic response and energy consumption under various operating conditions. Both the VBM- and propeller-actuated vehicles were evaluated. The results demonstrate that the proposed controller achieves an average energy consumption reduction of 22% compared to the next most efficient PID-based controller for the VBM-actuated vehicle, though with some impact on control performance.

## 1. Introduction

The ocean is home to a wide array of biological entities that require in-depth study and contains abundant natural resources such as oil and gas, which need to be responsibly extracted. Addressing environmental pollution in the ocean is also a crucial global issue today. However, ocean waters are heavily influenced by currents, waves, and other dynamic factors, making the underwater environment highly complex and potentially hazardous. As a result, tasks such as underwater pipeline inspections, offshore infrastructure upkeep, seabed and deep-sea exploration, collecting samples from marine environments, and monitoring and gathering data on ecological aquatic phenomena are very complex, time-consuming, and expensive. To tackle these challenges, it is crucial to design and develop underwater robots that meet high survivability standards while being user-friendly and capable of autonomous operation [1].

Depth control is critical in autonomous underwater vehicles (AUVs) due to its direct impact on the vehicle’s ability to perform precise and effective underwater tasks [2,3]. Accurate depth control ensures that AUVs can maintain stable navigation and positioning, which is essential for collecting reliable data in scientific research, conducting detailed underwater surveys, and executing complex industrial operations like pipeline inspections and offshore infrastructure maintenance. Furthermore, effective depth control is necessary for avoiding underwater obstacles and adapting to varying oceanographic conditions, such as changes in water density and current patterns. While precise depth control is essential, it must also consider the associated energy consumption, since energy efficiency in AUVs is vital for enhancing their operational effectiveness and extending mission duration. In this context, low-energy control strategies that can completely deactivate the actuators are of particular interest for applications requiring the sensor platform to maintain a minimal acoustic footprint. Examples of this situation occur whenever military [4,5], shipping [6,7], or seismic [8,9] activities should be traced, or, from a biology perspective, whenever whale population density should be identified [10] or sounds from fish [11], shrimp [12], or marine mammals [13] should be recorded, as they provide significant information on an ecosystem’s health. Also, sea surface wind speed [14] and ice shelf calving [15] can be monitored using underwater acoustics, for which it is important that the sensor platform has the minimum self-generated sounds.

One key topic in this area is the decision of using propellers or a variable buoyancy module (VBM) for depth control. In [16], a comparison between both actuators is made using a numerical example of an underwater glider. The authors of [16] conclude that propellers can match or even outperform the efficiency of a buoyancy engine. However, in the authors’ opinion, there are two shortcomings that might compromise the conclusions of the study [16]: (i) the force used to calculate the energy spent by the propeller-actuated glider does not seem comparable to the one used when considering a variable buoyancy system; (ii) the study does not include any hovering operations, in which a slight buoyancy offset may give a considerable advantage to a buoyancy engine over a propeller solution. Also, in this context, the authors of the present paper presented in [17] a detailed model of hydraulic and electromechanical variable buoyancy systems. These models were applied to numerous numerical examples based on typical mission profiles. The paper results demonstrated that using a VBS offers significant advantages over vertical propellers regarding energy consumption for hovering tasks. In fact, even at relatively shallow depths and shorter mission durations (contrary to what might be intuitively expected), the use of a VBS, particularly the electromechanical variant, proves more beneficial than relying on propellers.

Another interesting approach can be found in [18], where a hybrid passive buoyancy compensation system (HPBCS) for underwater gliders (UGs) is developed, integrating a spring accumulator, inflatable accumulator, and silicon oil bladder to address buoyancy variations. These passive elements are meant to compensate for the buoyancy change in the vehicle due to the balance between hull deformation and seawater density change with depth. This scheme increases efficiency because the active buoyancy change device does not have to spend energy compensating for the aforementioned buoyancy change. A mathematical model of the system is developed, and the design parameters are optimized using a genetic algorithm–tabu search (GA–TS) method. The performance of the HPBCS is evaluated through a case study on the Petrel-II glider, showing significant improvements in motion stability and energy efficiency compared to previous single-device methods. Although very valuable, this study is limited to energy savings due to the passive elements, not considering any gains that might come from using different control strategies in the active buoyancy changes. In another study [3], a high-accuracy buoyancy-actuated system (HA-BAS) for multimodal underwater vehicles (MUVs) is designed to optimize energy use and to provide precise depth control in deep-sea environments. The buoyancy module used in [3] is hydraulic and three different pump actuation schemes are explored. Motion simulations and sea trials confirm that two of those pump actuation schemes enhance energy savings. Although providing valuable results, the pump actuation schemes proposed in [3] appear to be based in an ad hoc procedure which might not be easily applied to other type of buoyancy control systems.

Another line of research within the topic of energy reduction is focused on the balance that must be struck between the high control effort needed for accurate depth control and the lower control effort required to conserve energy. This balance has been explored extensively in the literature.

In [19], a nonlinear backstepping depth controller for a hybrid (propeller- and buoyancy-driven) AUV is developed. The controller therein developed considers that a torpedo-shaped AUV has a low inertia and drag in the roll dynamic and can thus easily experience oscillatory behavior due to propeller torque or unknown disturbances. Simulation results show good control performance in both constant and variable depth references. Unfortunately, nothing is reported about energy consumption obtained with the proposed control law.

When considering the effect that the control law has on the energy consumption of the vertical motion of the vehicle, study [20] shows an interesting control approach simulating a human intelligent control (HSIC) and S-plane control. The controller is designed for the vertical motion of a long-range AUV (LRAUV). Although the LRAUV considered in [20] has several actuators (a stern thruster, shifting mass mechanism, a buoyancy adjustment mechanism, and a set of horizontal and vertical rudders), the study developed in [20] is focused only on two of those actuators: the horizontal rudders and the shifting mass mechanism. Both actuators change the value of the pitch angle, thereby affecting the depth of the vehicle which is assumed to travel at a constant horizontal speed. The controller developed in [20] includes an allocation strategy that dynamically decides which of the actuators is put in action, and a hybrid controller that switches the control structure according to the state of the system. For large depth errors, a bang–bang controller is used. When the depth error and the pitch angle are both small, the S-plane modal control [21] is adopted and finally, when depth error and pitch angle are within a certain range, the control action is kept constant. It is shown, through simulations, that by using the proposed control strategy, the energy consumption of each actuator is smaller than the energy consumption of the corresponding actuator under a single control mode. Unfortunately, no comparison with other control strategies is made in [20], so the actual energy gains obtained are unclear.

The authors of this study have previously conducted research analyzing the impact of various control laws on the energy consumption for depth control in underwater devices. In [2], the energy consumption of two actuation mechanisms for underwater platforms: variable buoyancy and propeller-actuated devices, was examined. Using a previously developed prototype, a detailed dynamic model was created to estimate energy consumption for both systems, incorporating nonlinearities such as saturations, sensor quantization, and actuator brake models. Several PID-based controllers were designed and tested through simulations. The results showed that variable buoyancy systems can drastically reduce energy consumption—by approximately 60%—for hovering operations compared to propeller-driven systems. However, this energy efficiency comes with a trade-off of greater depth control error, though variable buoyancy systems in [2] achieved faster settling times.

This paper expands the work in [2] with the following contributions: (i) it expands the model in [2] by considering the deformation of the vehicle hull with pressure; (ii) it provides a simple procedure, based on finite element analysis, to estimate such deformation; (iii) it presents a new buoyancy disturbance observer; (iv) it presents a new variable structure controller, developed for energy savings; and (v) it demonstrates that the proposed controller can further reduce energy consumption compared to the controllers presented in [2].

This paper is organized as follows: Section 2 presents the vehicle prototype and its model, including a detailed description of how the parameter expressing the vehicle deformation with pressure was estimated. Section 3 presents a new buoyancy disturbance observer, and Section 4, a new variable structure controller developed for low energy consumption. Section 5 presents several mission simulations, using both the controller developed in Section 4 and previous controllers developed in the literature. Finally, Section 6 draws the main conclusions of this work.

## 2. Prototype Description and Model

### 2.1. Prototype Description

**Figure 1:**
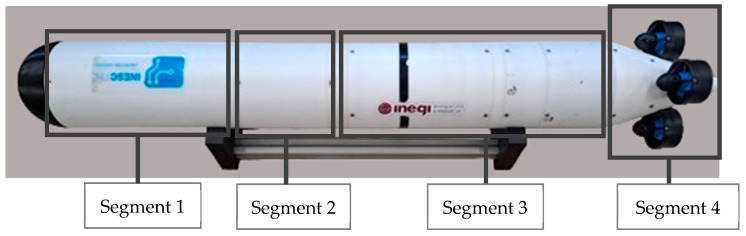
A picture of the prototype.

To investigate the potential benefits of a buoyancy-driven mechanism on energy efficiency, the authors developed a prototype featuring a VBM, as previously described in the literature [22] and presented in Figure 1. It should be noted that in standard operation, the prototype works with the main axis in the vertical direction. This prototype includes a main control unit (MCU—segment 1) for autonomous operation and is tailored for integration into autonomous underwater vehicles (AUVs) using modular building blocks or, alternatively, for standalone use as a buoy for vertical profiling or hovering tasks. In [2], a propeller module (PM—segment 4) was integrated into the prototype to allow for the comparison of energy consumption in real-world applications. The prototype also includes an intermediate section for floatation foam (segment 2) and the VBM (segment 3). Its overall dimensions are 1616 mm in length, 200 mm for the outer diameter, 180 mm for the inner diameter, and its dry weight is 36 kg. Segment 1 has a length of 400 mm, segment 2 has a length of 200 mm, segment 3 has a length of 616 mm, and segment 4 has a length of 300 mm. The black end cap seen at the left side of Figure 1 has a length of 100 mm. Buoyancy adjustment is achieved by pumping seawater via a diaphragm-sealed piston mechanism [22] driven by an electric motor coupled to a mechanical transmission and spindle. This VBM enables a total volume change of approximately ±350 cm^3^ and can operate down to a depth of 100 m. This prototype, which will be used in this work without any hardware changes relative to the one presented in [2], incorporates two sensors which are standard for this type of module: (i) a pressure sensor, which is used to determine the depth of the vehicle and (ii) a position sensor to measure the position of the diaphragm-sealed piston mechanism. This second sensor allows the estimation of the buoyancy force as it enables the measurement of the variable volume. Only these two sensors are required for the controllers developed in subsequent sections.

The main focus of this work is to introduce a novel variable structure controller to reduce the energy consumption of the prototype, along with a disturbance observer to assist in controlling tasks by estimating the disturbances affecting the prototype. To this end, in Section 2.2. the model of the prototype required for this purpose is presented.

### 2.2. Prototype Model

**Figure 2:**
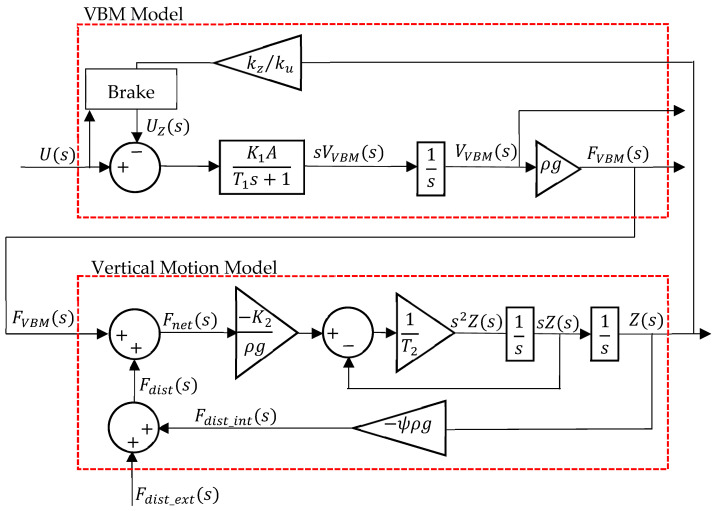
Prototype model.

The prototype model comprised the combination of the VBM model and the vertical motion model, as developed in [23], and is presented in Figure 2.

In Figure 2, $K_{1}$ and $T_{1}$ are the actuator velocity dynamics steady-state gain and time constant, respectively, that were experimentally identified in [22], and A is the area of the piston in contact with sea water. When the control action U=0, the brake is engaged, so the equivalent depth voltage $U_{Z}$ is zero. If U≠0, the brake is disengaged, so $U_{Z}=k_{z}⁄k_{u}Z$ to account for the influence of pressure due to depth Z on the actuator motion. The ratio $k_{z}/k_{u}$ was determined in [2] and models the torque caused on the motor due to the force exerted by the outside pressure, as well as the motor stall torque at the applied voltage level. $F_{net}$ is the balance between the actual VBM buoyancy force $F_{VBM}$ (generated by the VBM buoyancy volume $V_{VBM}$), and the disturbances acting on the prototype $F_{dist}$, whether internal ($F_{dist\_int}$) or external ($F_{dist\_ext}$). $K_{2}$ and $T_{2}$ are the depth dynamics parameters identified in [23]. The parameters ρ and g are the water mass per unit volume (assumed to be constant, $\rho =1000\;\mathrm{kg}/m^{3}$) and the acceleration of gravity ($g=9.8\;{\mathrm{ms}}^{-2}$), respectively. Parameter ψ expresses the loss of volume per meter of depth due to hull deformation and will be determined in Section 2.3. This loss of volume will result in a disturbance force $F_{dist\_int}$.

### 2.3. Estimation of Parameter ψ

During an underwater mission, an AUV experiences the effects of pressure due to the water column height. This effect happens in all submerged bodies, but it is especially relevant in sealed hollow bodies with a compressible fluid inside. In fact, when a solid body is submerged, the water pressure is evenly distributed along the whole body’s surface area, leading to a pressure equilibrium state in which the main load is one of compression. Though this effect may be relevant for the submerged volume of a solid body, it is reasonably safe to assume that its impact is minimal when compared to a sealed volume with a compressible fluid on the inside, such as air. In this situation, the equilibrium of pressure acting on the outside and inside of the hollow body may not be achieved without a significant amount of deformation, which is heavily dependent on the body material stiffness. Considering this, the deformation of the hull of an AUV can have serious consequences on its buoyancy, particularly in dry segments of the vehicle. To assess the effect of pressure on the volume occupied by the dry segments of the prototype, simplified 3D models for the VBM and MCU segments were created using Solidworks (release 2020). The VBM model consists of a hollow cylinder of uniform material. Simulations using the finite elements method were then run on the VBM 3D model considering the following conditions:

- Material: POM acetal copolymer;
- Fixtures:
  1. Each external edge of the cylinder is not allowed to rotate about the cylinder longitudinal axis;
  2. One of the external edges is not allowed to move along the longitudinal axis;
- External loads: pressure along the whole outside surface area of the cylinder.

**Figure 3:**
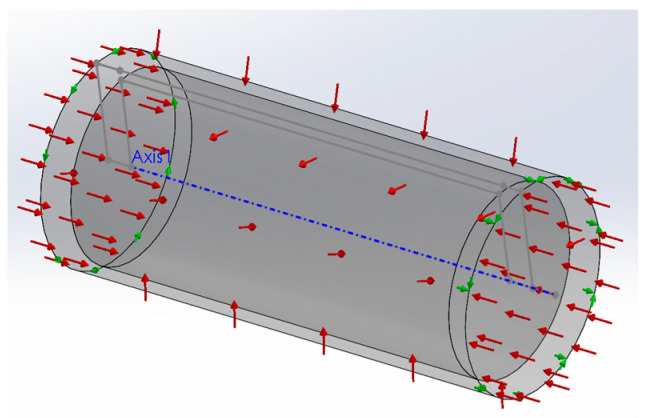
VBM 3D model with fixtures and loads for simulation.

The 3D model with the applied fixtures and loads is presented in Figure 3.

**Figure 4:**
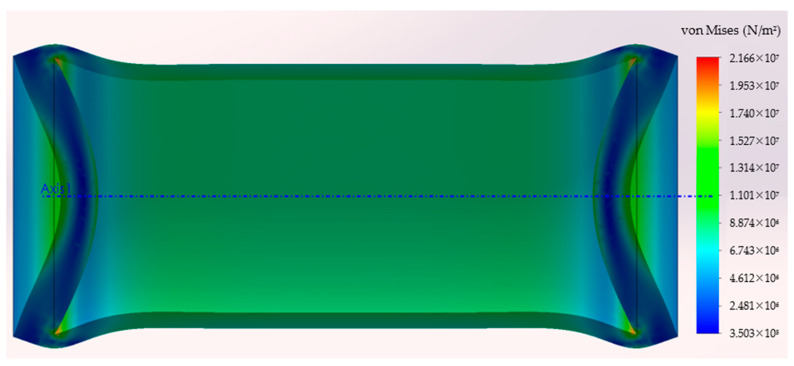
Deformed shape of the VBM module and von Mises stress.

In Figure 3, the green arrows represent fixtures, and the red ones represent the loads, as described in the conditions above. The simulation was run with different pressure levels (2.5, 5, 7.5, 10 bar) to simulate the effect of water depths up to 100 m. Figure 4 presents the deformed shape of the 3D model subjected to a 10-bar pressure load. In this figure, the deformations are represented with an amplification of 40 times in order to be visible. The von Mises stress is represented with a jet colormap. The true deformed shape of the model was then imported back to SolidWorks as a new part, and the new closed volume was calculated ($Vol_{p}$). Table 1 presents the results of the VBM 3D model volume under different pressure levels and the difference to the ambient pressure volume $Vol_{0}.$

**Figure 5:**
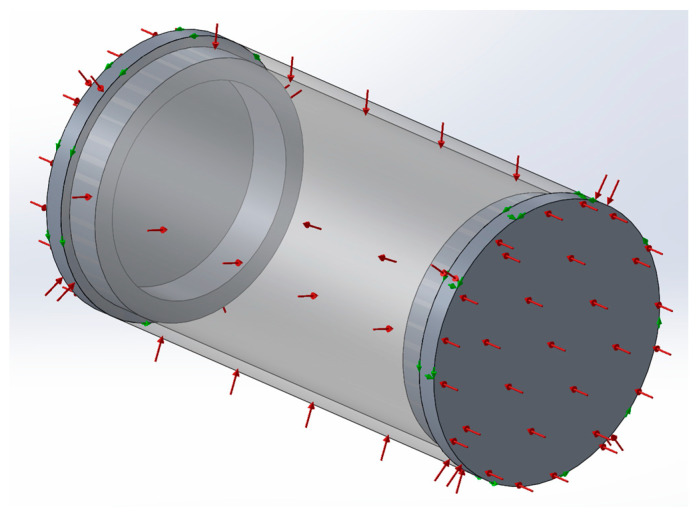
MCU 3D model with fixtures and loads for simulation.

The results presented in Table 1 show that the loss of volume due to pressure is approximately linear in the simulated pressure range. This validates the use of a parameter ψ to express the loss of volume per meter depth. An identical process was followed with the MCU. In the case of the MCU 3D model, however, an assembly of the acrylic cylinder body and two aluminum flanges was considered. The MCU 3D model is presented in Figure 5.

**Figure 6:**
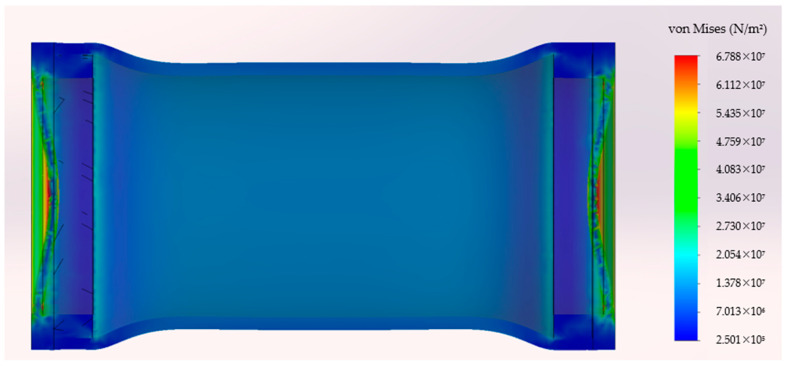
Deformed shape of the MCU model and von Mises stress.

The deformed shape (amplified by a factor of 40 times) and the von Mises stress on the 3D model subjected to a 10-bar pressure load is shown in Figure 6. As performed with the VBM model, the true deformed shape was reimported to SolidWorks as a new part, and the new volume calculated. It was found that for a 10-bar pressure, the MCU loss of volume would be about 7 cm^3^. Finally, the parameter ψ, the loss of volume per meter depth, was calculated as the sum of the volume loss of the VBM (${\left.\left(Vol_{0}-Vol_{p}\right)\right|}_{VBM}$) and the volume loss of the MCU ${\left.\left(Vol_{0}-Vol_{p}\right)\right|}_{MCU}$, at 100 m (10 bar of pressure):

(1) $$\psi =\frac{{\left.\left(Vol_{0}-Vol_{p}\right)\right|}_{VBM}+{\left.\left(Vol_{0}-Vol_{p}\right)\right|}_{MCU}}{100}=0.34\;\left[\frac{cm^{3}}{m}\right]=3.4\cdot 10^{-7}m^{3}/m$$

## 3. Buoyancy Disturbance Observer

**Figure 7:**
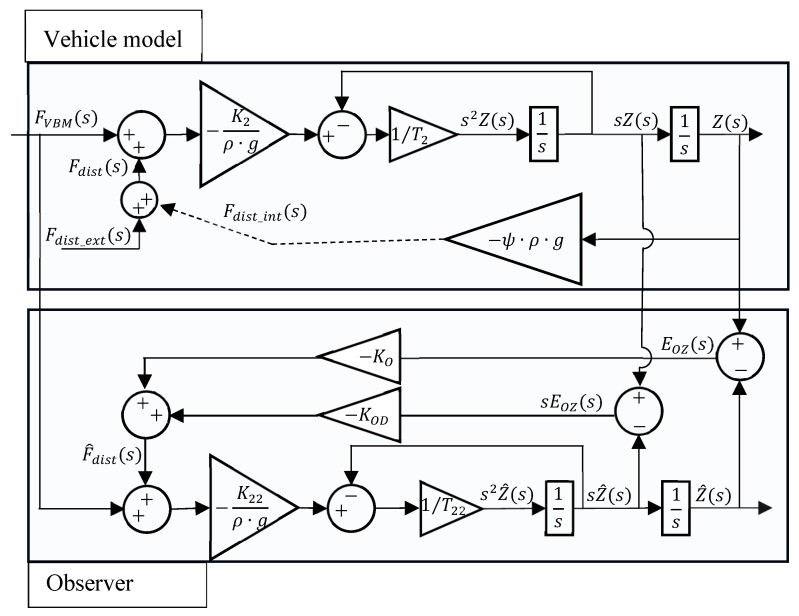
Block diagram of the vehicle model and observer.

The buoyancy disturbance observer is based on the block diagram in Figure 7. The vehicle model in the vertical direction, shown in the upper part of Figure 7, is a second order system with parameters $K_{2}$, $T_{2}$, and ψ. $K_{2}$ and $T_{2}$ have been identified in [23] and ψ has been estimated in Section 2 of this paper. The system model used for the observer is also second-order, but it will be assumed in the following that its parameters might not be fully known. This allows the study of the effect of this uncertainty in the observer performance. It will be considered that $K_{22}$ is an estimate of $K_{2}$, while $T_{22}$ is an estimate of $T_{2}$, such that:

(2) $$K_{22}=K_{2}+\xi_{K}$$

and

(3) $$T_{22}=T_{2}+\xi_{T}$$

where $\xi_{K}$ is the error in the $K_{22}$ parameter and $\xi_{T}$ is the error in the $T_{22}$ parameter.

For the purpose of the disturbance observer synthesis, the disturbance force ($F_{dist}$) that will be observed (${\hat{F}}_{dist}$) is considered to be composed of two parts: an external one $(F_{dist\_ext}$) caused, for instance, by dropping or collecting a weight, and an internal one $(F_{dist\_int}$), caused by the vehicle deformation due to pressure.

Based on Figure 7, the error $E_{OZ}\left(s\right)$ between the vehicle depth $Z\left(s\right)$ and the observer depth estimation $\hat{Z}\left(s\right)$ can be written as

(4) $$E_{OZ}\left(s\right)=\left(\frac{-K_{2}\left(T_{22}s^{2}+s\right)+K_{22}\left(T_{2}s^{2}+s\right)}{\left(\rho \cdot g\left(T_{22}s^{2}+s\right)+K_{22}K_{O}+K_{22}K_{OD}s\right)\left(T_{2}s^{2}+s\right)}\right)F_{VBM}\left(s\right)-\frac{\left(T_{22}s^{2}+s\right)}{\left(\rho \cdot g\left(T_{22}s^{2}+s\right)+K_{22}K_{O}+K_{22}K_{OD}s\right)}\frac{K_{2}}{\left(T_{2}s^{2}+s\right)}F_{dist}\left(s\right)$$

The disturbance estimation error $E_{dist}\left(s\right)$ can be written as

(5) $$E_{dist}\left(s\right)=F_{dist}\left(s\right)-\left({\hat{F}}_{dist}\left(s\right)\right)=F_{dist}\left(s\right)+E_{OZ}\left(s\right)K_{O}+E_{OZ}\left(s\right)sK_{OD}$$

Replacing (4) in (5) leads to (6), the transfer functions between $F_{VBM}\left(s\right)$ and the disturbance observer error $E_{dist}\left(s\right)$ and between the disturbance force $F_{dist}\left(s\right)$ and $E_{dist}\left(s\right)$ can be written as

(6) $$E_{dist}\left(s\right)=\left(\frac{\left(\rho \cdot g\left(T_{22}s^{2}+s\right)+K_{22}K_{O}+K_{22}K_{OD}s\right)\left(T_{2}s+1\right)-\left(T_{22}s+1\right)K_{2}\left(K_{O}+sK_{OD}\right)}{\left(\rho \cdot g\left(T_{22}s^{2}+s\right)+K_{22}K_{O}+K_{22}K_{OD}s\right)\left(T_{2}s+1\right)}\right)F_{dist}\left(s\right)\;\;\;\;\;\;\;\;+\left(\frac{-K_{2}\left(T_{22}s+1\right)+K_{22}\left(T_{2}s+1\right)}{\left(\rho \cdot g\left(T_{22}s^{2}+s\right)+K_{22}K_{O}+K_{22}K_{OD}s\right)\left(T_{2}s+1\right)}\right)\left(K_{O}+sK_{OD}\right)F_{VBM}\left(s\right)$$

The dynamics between the disturbance $F_{dist}\left(s\right)$ and the observer error $E_{dist}\left(s\right)$ can be rewritten as

(7) $$E_{dist}\left(s\right)=\left(1-\frac{T_{22}K_{2}K_{OD}s^{2}+T_{22}K_{2}K_{O}s+K_{2}K_{OD}s+K_{2}K_{O}}{\rho gT_{2}T_{22}s^{3}+\left(\rho gT_{22}+T_{2}\rho g+K_{22}K_{OD}T_{2}\right)s^{2}+\left(\rho g+K_{22}K_{OD}+K_{22}K_{O}T_{2}\right)s+K_{22}K_{O}}\right)F_{dist}\left(s\right)$$

Analyzing Equation (7), it can be concluded that the observer dynamics cannot be fully imposed as they present third-order dynamics, and the observer only has two adjustable parameters. The transfer function (7) can nevertheless be used to adequately tune the observer dynamic response since its poles are given by

(8) $$s=-\frac{1}{T_{2}}\vee s=\frac{-\rho g-K_{22}K_{OD}\pm \sqrt{{\left(\rho g+K_{22}K_{OD}\right)}^{2}-4K_{22}K_{O}T_{22}\rho g}}{2T_{22}\rho g}$$

A particular case of Equation (7) occurs when there is not any error in the observer parameters $K_{22}$ and $T_{22}$. In this case, the disturbance of observer dynamics does not depend on the force $F_{VBM}\left(s\right)$, and the transfer function between $F_{dist}\left(s\right)$ and $E_{dist}\left(s\right)$ simplifies to Equation (9):

(9) $$E_{dist}\left(s\right)=\frac{\rho \cdot g\left(T_{22}s^{2}+s\right)}{\rho gT_{22}s^{2}+\left(\rho g+K_{22}K_{OD}\right)s+K_{22}K_{O}}F_{dist}\left(s\right)$$

The poles in this situation are given by

(10) $$s=\frac{-\;\rho g-K_{22}K_{OD}\pm \sqrt{{\left(\rho g+K_{22}K_{OD}\right)}^{2}-4K_{22}K_{O}T_{22}\rho g}}{2T_{22}\rho g}$$

As seen in (10), when the observer parameters exactly match the system parameters, the observer poles are the complex poles of (8), which can be placed according to the values of $K_{O}$ and $K_{OD}$. The upper pole of (8) appears only when the observer parameters are different from the system parameters.

It is now possible to determine the steady-state error of the observer for different $F_{dist}$ and $F_{VBM}$ inputs. Considering a step of amplitude $A_{1}$ for $F_{dist}$ and a step of amplitude $A_{2}$ for $F_{VBM}$, the steady-state error can be calculated by the summation of the errors due to each input, leading to

(11) $$\underset{s\to 0}{\lim}\;s\left(E_{dist}\left(s\right)\right)=\left(\frac{\xi_{K}}{K_{2}+\xi_{K}}\right)A_{1}+\left(\frac{\xi_{K}}{K_{2}+\xi_{K}}\right)A_{2}$$

The result of Equation (11) shows that the steady-state error of a constant input becomes smaller as the error in the $K_{2}$ parameter becomes smaller. If this error becomes zero, then the observer can predict exactly a constant disturbance at a steady state. Note also that the result of Equation (11) does not depend on $\xi_{T}$ since the time constant does not affect the steady-state error.

Considering now a ramp of slope $R_{1}$ for $F_{dist}$ and of slope $R_{2}$ for $F_{VBM}$, the steady-state error can be calculated by the summation of the errors due to each input, leading to

(12) $$\underset{s\to 0}{\lim}\;sE_{dist}\left(s\right)=\underset{s\to 0}{\lim}\left(\left(\frac{\xi_{K}}{K_{2}+\xi_{K}}\right)\frac{R_{1}}{s}+\left(\frac{\xi_{K}}{K_{2}+\xi_{K}}\right)\frac{R_{2}}{s}\right)=\infty$$

Analyzing Equation (12), the error of a ramp disturbance is infinite, i.e., the observer cannot adequately track a ramp disturbance. In the particular case when there is not any error in the observer parameters $K_{22}$ and $T_{22}$, it is clear from (9) that $F_{VBM}$ has no influence on the observer tracking capability. In this case, the steady-state error of the observer to an $F_{dist}$ ramp input with a slope $R_{3}$ can be obtained using Equation (9) as

(13) $$\underset{s\to 0}{\lim}\;sE_{dist}\left(s\right)=\underset{s\to 0}{\lim}\;s\left(\frac{\left(\rho \cdot g\right)\left(T_{22}s^{2}+s\right)}{\rho gT_{22}s^{2}+\left(\rho g+K_{22}K_{OD}\right)s+K_{22}K_{O}}\right)\frac{R_{3}}{s^{2}}=\frac{\rho g}{K_{22}K_{O}}R_{3}$$

An analysis of Equation (13) shows that when the model parameters are known, the observer is able to track a ramp input, although with an error which decreases with the increase in $K_{O}.$

According to Equation (8), it is not possible to fully impose a dynamic for the observer, since one of its poles is exclusively a function of the time constant $T_{2}$ and therefore, its position is set. As for the other two poles, they will depend on the values chosen for $K_{O}$ and $K_{OD}$. A common strategy when performing pole placement is to choose all real poles to minimize undesired oscillatory behavior. Therefore, the radicand in Equation (8) must be equal to or greater than zero:

(14) $${K_{22}}^{2}{K_{\mathrm{OD}}}^{2}+2\rho gK_{22}K_{OD}+{\left(\rho g\right)}^{2}-4K_{22}K_{O}T_{22}\rho g\geq 0$$

Solving for $K_{OD}$ to determine the roots of the polynomial:

(15) $$K_{OD}=-\frac{\rho g}{K_{22}}\pm 2\sqrt{\frac{K_{O}T_{22}\rho g}{K_{22}}}$$

Since, from Equation (8), $K_{OD}\geq -\rho g/K_{22}$ to ensure stability of the observer, there is only one possible root:

(16) $$K_{OD}=-\frac{\rho g}{K_{22}}+2\sqrt{\frac{K_{O}T_{22}\rho g}{K_{22}}}$$

For the inequality in (14) to be ensured, and since ${K_{22}}^{2}>0$, the following condition must be met:

(17) $$K_{OD}=-\frac{\rho g}{K_{22}}+2\sqrt{\frac{K_{O}T_{22}\rho g}{K_{22}}}\geq 0$$

Solving for $K_{O}$ in Equation (17) leads to

(18) $$K_{O}\geq \frac{\rho g}{4T_{22}K_{22}}$$

An analysis of expressions (16) and (18) shows that when the observer gain $K_{O}$ takes the minimum value of Equation (18), ($K_{O}=\frac{\rho g}{4T_{22}K_{22}}$), then it follows by Equation (16) that $K_{OD}=0$. Replacing these two values in Equation (8), the critical damping situation occurs, and the poles overlap at $s=-1/\left(2T_{22}\right).$

## 4. Controllers

The depth control of the prototype can generically be performed using several different approaches. In [2], the authors proposed two control architectures based on PID family controllers: a single-depth controller for the PM and a cascaded control strategy consisting of a depth controller and a volume controller for the VBM. Among the controllers tested in [2], the structures that led to the smallest energy consumption were selected for datum comparison in this work:

- Structure 3b: a PID depth controller and a PI volume controller (VBM);
- Structure 7b: an I-PID depth controller and a PI volume controller (VBM);
- Structure 11b: an I-PD depth controller (PM).

**Figure 8:**
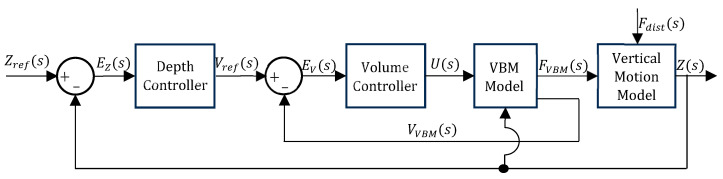
Block diagram for structures 3b and 7b.

**Figure 9:**
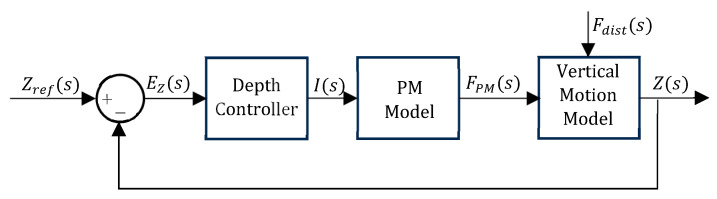
Block diagram for structure 11b.

Figure 8 presents the block diagram for structures 3b and 7b. In Figure 8, $Z_{ref}$ is the target depth reference and $E_{Z}$ is the depth error, whereas $V_{ref}$ is the target volume reference and $E_{V}$ is the volume error. Figure 9 shows the block diagram for structure 11b, where I is the control action and $F_{PM}$ is the PM buoyancy force.

The full detail of each controller structure can be found in [2]. This paper introduces a new variable structure controller which will be detailed in the next section.

### Proposed Variable Structure Controller

**Figure 10:**
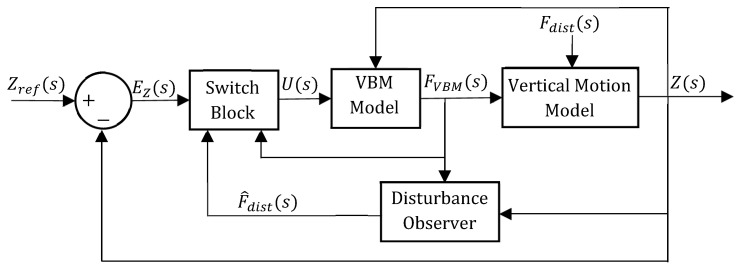
VSC system block diagram.

To minimize the energy consumption of the VBM, a novel variable structure controller (VSC) was devised, as presented in Figure 10. The underlying idea behind the VSC is to make use of the systems’ inherent dynamics, whenever it is favorable to the prototype depth control. In other words, switching on the VBM is only required whenever the prototype is moving away from the depth target reference. When the prototype is moving in the direction that minimizes the depth error, it should be switched off. In this way, the VBM is expected to be switched off for a considerable amount of time throughout the mission, therefore saving energy.

**Figure 11:**
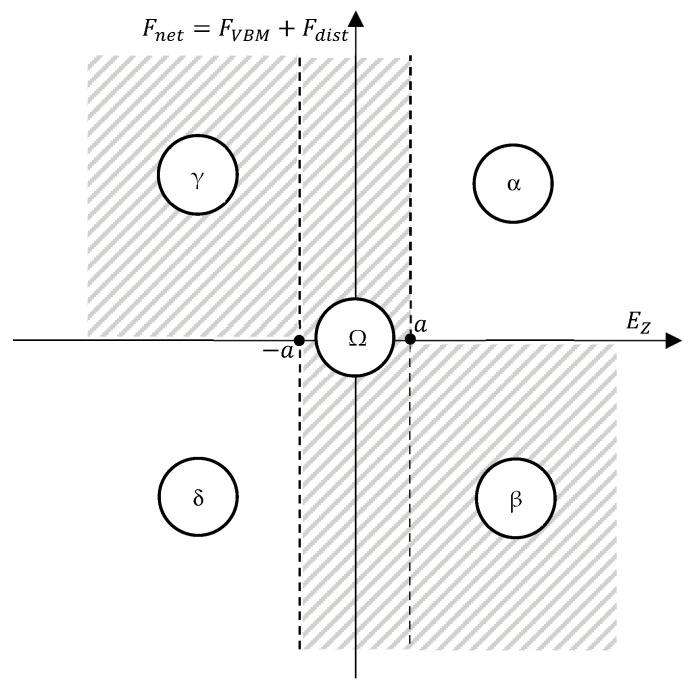
Schematic of the switch block decision process.

The switch block, detailed below, is responsible for deciding whether it is necessary to switch on the VBM or not. Figure 11 presents a schematic to explain the switch block decision process.

In the shaded regions of the schematic in Figure 11, the VBM is switched off, and in the remaining regions, it is switched on. If there are no disturbances present, $F_{VBM}$ is sufficient to characterize the prototype depth acceleration, since it is the only force that can change the prototype vertical motion equilibrium. In real-world conditions, there are countless possible disturbances that may occur during an underwater mission, such as load lifting, buoyancy trimming errors, and hull deformation, among others. This means that it is possible for a situation to occur where $F_{VBM}$ should lead to an expected output in terms of prototype acceleration, but the existence of $F_{dist}$ does not lead to the expected prototype movement. To take into account disturbances, the vertical axis of Figure 11’s schematic represents the net force $F_{net}$ ($F_{net}=F_{VBM}+F_{dist}$). Parameter a is a depth deadband, meaning there is an admissible depth error in which the controller is switched off ($\left|E_{Z}\right|<a⟹U=0$). Considering the system starts in region α, the depth error $E_{Z}$ and net force $F_{net}$ are positive ($E_{Z}>0,F_{net}>0$). In this situation, the target depth reference is deeper than the current depth ($Z_{ref}>Z$) but the positive buoyancy would lead to a decrease in depth, which would increase $e_{Z}$. For this reason, the VBM should be switched on in this case, so that $U=-sign\left(E_{Z}\right)\cdot U_{switch}$. Since U will directly act on the derivative of $V_{VBM}$ and so, on the derivative of $F_{VBM}$ (please check Figure 2), the value of $F_{net}$ will eventually become negative and the depth error will eventually decrease. A similar line of thought can be followed for region δ of Figure 11, while keeping in mind that in this region, the depth error and the net force are both negative. In regions β and γ, the depth error and the net volume have opposite signs; therefore, despite the VBM being turned off, the natural dynamics of the system will inevitably lower the error, leading the system to region Ω. The system cannot remain at a steady state inside region Ω, so it will eventually move to the other regions. From the description above, it is possible to infer that parameter a has a direct influence on the steady-state error.

Table 2 presents the different switch block decisions and control actions U according to each region in the schematic of Figure 11.

## 5. Simulation Results

In this section, the VSC performance presented in Section 4 will be assessed. To that end, the performance of the VSC will be tested against (i) the PID-type controllers presented in [2] leading to the lowest energy consumption of the VBM (controllers 3b and 7b of [2]) and (ii) the PID-type controller leading to the lowest energy consumption of the prototype actuated by propellers (controller 11b of [2]). To simulate the VSC, it is also necessary to determine adequate gains for the disturbance observer. In the simulations, it was assumed there was no error in the estimation of parameters $K_{2}$ and $T_{2}$. The observer gains were chosen so that all poles are real. Table 3 presents the values chosen for the simulation. Table 4 lists the observer pole locations.

The controller gains for the controllers presented in [2] are the same as the ones used in [2]. Since those controller gains were chosen to minimize the energy consumption of the prototype, the same premise was considered for the VSC. As such, the parameters for the VSC were experimentally tuned to keep a low energy consumption while maintaining an acceptable time response. The chosen parameters were $U_{switch}$ = 8 V and a = 0.1 m.

**Figure 12:**
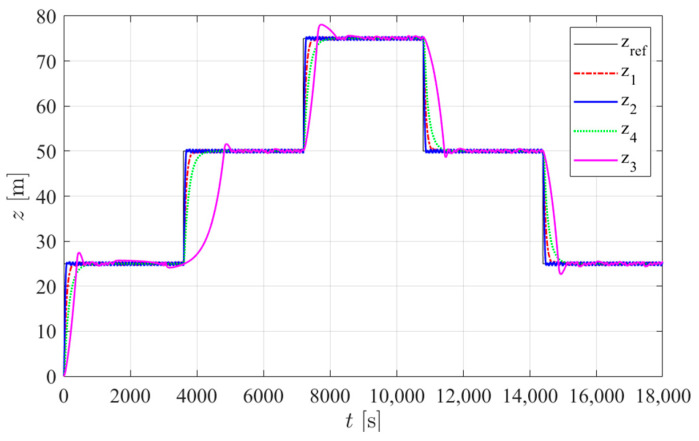
Simulated prototype depth for each controller in type 1 trials: z_ref_ is the reference position and z*_j_* is the depth obtained with controller *j* in Table 5.

**Figure 13:**
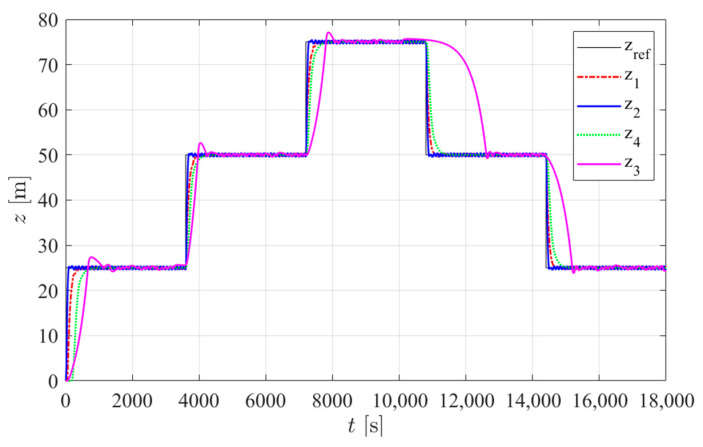
Simulated prototype depth for each controller in type 2 trials: z_ref_ is the reference position and z*_j_* is the depth obtained with controller *j* in Table 5.

**Figure 14:**
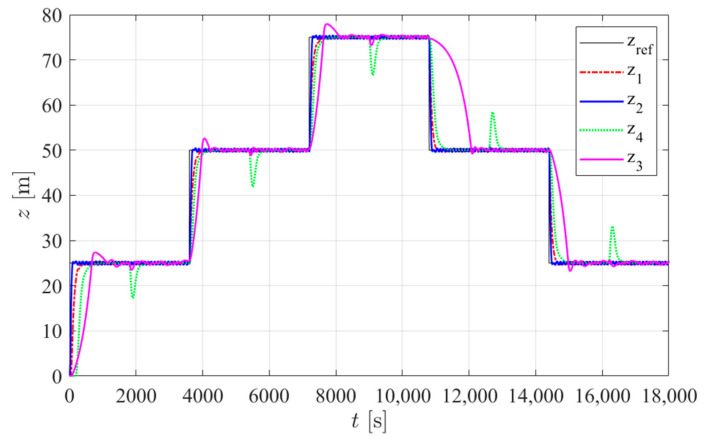
Simulated prototype depth for each controller in type 3 trials: z_ref_ is the reference position and z*_j_* is the depth obtained with controller *j* in Table 5.

The simulation trials were run in Matlab Simulink (R2019a) with the same depth target reference signal and the same external buoyancy force disturbances considered in [2]. Three disturbance scenarios were applied: in type 1 trials, no disturbance was considered. In type 2 trials, a constant disturbance of 1.75 N was added. In type 3 tests, the disturbance increased in discrete 0.35 N steps: starting from 1.75 N at time *t* = 0 s, it increased by 0.35 N at *t* = 1800 s, then increased by 0.35 N every 1800 s until it reached 2.8 N. At *t* = 12,600 s, it then decreased by 0.35 N every 1800 s until it reached 2.1 N at *t* = 16,200 s. This disturbance value was then held until *t* = 18,000 s. In this work, the internal disturbance of the hull deformation was added with the parameter ψ = 3.4 × 10^−7^ m^3^/m, as estimated in Section 2.3. The energy consumption results for each controller are presented in Table 5. Figure 12, Figure 13 and Figure 14 show the simulated prototype depth during the three tests with each controller.

To evaluate the performance in step *i* (*i* = 1:5) of the controller *j* (*j* = 1:4), the performance metrics outlined in [2] and listed below were used.

1. Overshoot: $Mp_{i,j}$
2. Maximum error magnitude $\left|Ezm_{i,j}\right|$: this metric is defined as the maximum error after the third inflection point of the prototype for each step i and controller j.
3. Absolute settling time $tssa_{i,j}$: the time required for the prototype to reach and stay within its $\left|Ezm_{i,j}\right|$ band.
4. Relative settling time $tssr_{i,j}$: the time required for the prototype to reach the widest band across all control structures ($max_{j}\left(\left|Ezm_{i,j}\right|\right)$).
5. Maximum error after new external disturbance: $Ezd_{i,j}$.

Table 6 presents the average values of these metrics for all steps *i* and each controller *j*.

Considering the results presented in Table 5 and Table 6, it is clear that when considering the average energy consumption for all trials, the prototype actuated by controller 3 leads to the lowest values, requiring 22% less energy when compared to the next best-performing controller (controller 2). However, it yields the worst control performance, since on average, it leads to the highest overshoot, error magnitude, and relative settling time. In performance terms, it could be argued that controller 4 is the best, given the minimal average overshoot and error magnitude. Nevertheless, its energy consumption is the highest in the type 2 and 3 trials and it is very slow, with the highest average absolute settling time. This high average absolute settling time is also relevant to understand this combination’s high error after a new external disturbance, since it takes more time for the controller to react to the disturbance, during which the prototype depth error is increasing. The most balanced combination may be controller 1, since it leads to a low average overshoot, error magnitude, error after new external disturbance, and acceptable average absolute and relative settling times. In comparison, with a similar energy consumption, controller 2 leads to a bigger average overshoot and error magnitude. However, it is also the solution with the lowest average absolute and relative settling times.

Finally, the effectiveness of each controller in ensuring the sensor platform’s viability for underwater acoustic measurements was evaluated. This evaluation involved counting the duration each controller remained switched off for at least 60 s. Table 7 presents the accumulated total time for each trial and Table 8 shows the percentages of the total running time for each trial. It was observed that the controller proposed in this work remained switched off for at least 60 s for more than 75% of each trial duration. This indicates that the proposed controller is highly suitable for applications requiring a minimal acoustic signature. Notably, these results are comparable to those obtained for gliders (approximately 87% off time in [24]). However, while the trials in [24] were based on sawtooth-profiled trajectories, this study employed hovering operations, which pose greater challenges in keeping the actuators switched off, especially in the presence of disturbances.

Finally, it should be remarked that (i) controller 1 presents much less switched-off time than the proposed controller and (ii) controllers 2 and 4 could not be applied whenever the sensor platform was used for acoustic measurements, as they never switch off.

## 6. Conclusions

This paper focused on the development of a novel variable structure controller for depth control of an autonomous underwater device prototype using a variable buoyancy module. The parameters of the prototype linear model were presented and the prototype hull deformation due to pressure was estimated using a finite element method. The presence of possible disturbances acting on the prototype, such as hull deformation, led to the need to estimate said disturbances. For this reason, a disturbance observer was developed. The estimation error was analyzed according to the input disturbance and system parameter uncertainty. Additionally, the location of the observer poles was determined.

Considering the prototype linear model, a plane of state-dependent variables was defined and split into different regions. The proposed variable structure controller is switched on when the system’s natural dynamics are unfavorable (when the vehicle will tend to move away from the desired reference) and is switched off when the system’s natural dynamics are favorable (when the vehicle will tend to move toward the desired reference). A detailed analysis of the controller was performed to determine the necessary switching control action that guarantees that the system is driven to the desired reference.

The proposed controller was tested through a simulation along with PID-based controllers to evaluate their dynamic response and energy consumption across different operation conditions. Both VBM- and propeller-actuated vehicles were considered. The findings indicate that the proposed controller requires 22% less average energy consumption compared to the next best-performing controller (a PID-based controller used on the VBM-actuated vehicle). However, this energy reduction comes with a significant cost in terms of control performance in most of the proposed metrics. It can nevertheless be of important use whenever the mission only requires coarse-performance hovering tasks. An interesting feature of the proposed controller is that it is switched off for long periods of time, thereby being very silent, which is useful for underwater acoustic monitoring.

Future work will focus on sea trials of the proposed controller. Also, the development of controllers which can further reduce the time during which the system is switched on to reduce the acoustic trace of the sensor platform, will be pursued.

## Figures and Tables

**Table 1:** Volume of the VBM 3D model under different pressure levels.

$p\;\left[\mathrm{bar}\right]$	Depth [m]	$Vol_{p}\;\left[cm^{3}\right]$	$Vol_{0}-Vol_{p}\;\left[cm^{3}\right]$
0	0	14,279	0
2.5	25	14,272	7
5	50	14,265	14
7.5	75	14,259	20
10	100	14,252	27

**Table 2:** Switch block decision table.

Regions	$E_{Z}$	$F_{net}$	Decision	Control Action U
Ω	$-a<E_{Z}<a$	$F_{net}$	OFF	0
α	$E_{Z}>a$	$F_{net}>0$	ON	$-U_{switch}$
β	$E_{Z}>a$	$F_{net}<0$	OFF	0
γ	$E_{Z}<-a$	$F_{net}>0$	OFF	0
δ	$E_{Z}<-a$	$F_{net}<0$	ON	$U_{switch}$

**Table 3:** Disturbance observer minimum and chosen gains.

Gain	Value	Unit
$K_{O}$	5	Nm^−1^
$K_{OD}$	100	Nsm^−1^

**Table 4:** Disturbance observer poles.

Pole	Location
$s_{1}$	−0.0505
$s_{2}$	−2.2466

**Table 5:** Energy consumption results for the simulated trials.

Actuating Device	Controller		Energy [kJ]		
	*j*	Structure	Type 1	Type 2	Type 3
VBM	1	PID + PI (3b in [2])	46.9	45.8	43.9
	2	I-PD + PI (7b in [2])	41.8	42	42
	3	VSC	32.3	32.8	33.2
PM	4	I-PD (11b in [2])	29.4	53.8	68.6

**Table 6:** Performance metrics for each control structure.

			Type 1 Trials				
Actuating Device	Controller		$\overset{5}{\underset{i=1}{\sum }}Mp_{i,j}/5$ [%]	$\overset{5}{\underset{i=1}{\sum }}Ezm_{i,j}/5$ $\left[m\right]$	$\overset{5}{\underset{i=1}{\sum }}tssa_{i,j}/5$ $\left[s\right]$	$\overset{5}{\underset{i=1}{\sum }}tssr_{i,j}/5$ $\left[s\right]$	$\overset{5}{\underset{i=1}{\sum }}Ezd_{i,j}/5$ $\left[m\right]$
	*j*	Structure					
VBM	1	PID + PI (3b in [2])	0.8	0.391	411.1	260.6	−
	2	I-PD + PI (7b in [2])	1.6	0.502	77.8	76.4	−
	3	VSC	8.8	0.65	867.5	867.5	−
PM	4	I-PD (11b in [2])	0	0.001	1476.8	552.4	−
			**Type 2 Trials**				
VBM	1	PID + PI (3b in [2])	1.1	0.385	413.7	267.5	−
	2	I-PD + PI (7b in [2])	1.4	0.509	124.1	78.2	−
	3	VSC	7.5	0.66	1098.9	1098.9	−
PM	4	I-PD (11b in [2])	0	0.001	1162.4	480.2	−
			**Type 3 Trials**				
VBM	1	PID + PI (3b in [2])	1.1	0.386	433.5	280.2	0.635
	2	I-PD + PI (7b in [2])	1.4	0.506	126.7	102.7	0.776
	3	VSC	8.5	0.6	973.3	963.5	1.14
PM	4	I-PD (11b in [2])	0	0.001	1107.6	470.8	8.185

**Table 7:** Switched-off time for each trial.

Actuating Device	Controller		Accumulated Time [s]		
	*j*	Structure	Type 1	Type 2	Type 3
VBM	1	PID + PI (3b in [2])	562	452	369
	2	I-PD + PI (7b in [2])	0	0	0
	3	VSC	13,914	13,793	13,613
PM	4	I-PD (11b in [2])	0	0	0

**Table 8:** Switched-off time for each trial (percentages of the total running time for each trial).

Actuating Device	Controller		Switched-Off Percentage of the Total Running Time [%]		
	*j*	Structure	Type 1	Type 2	Type 3
VBM	1	PID + PI (3b in [2])	3.1%	2.5%	2.0%
	2	I-PD + PI (7b in [2])	0%	0%	0%
	3	VSC	77.3%	76.6%	75.6%
PM	4	I-PD (11b in [2])	0%	0%	0%

## Data Availability

Data are contained within the article.

## Nomenclature

a	Depth deadband [m]
A	Area of the piston in contact with sea water [m^2^]
$E_{dist}$	Disturbance observer error [N]
$E_{OZ}$	Disturbance observer depth error [m]
$E_{Z}$	Depth control error [m]
$F_{dist}$	Disturbance forces [N]
${\hat{F}}_{dist}$	Estimate of disturbance forces [N]
$F_{dist\_ext}$	External disturbance forces [N]
$F_{dist\_int}$	Internal disturbance forces [N]
$F_{VBM}$	Variable buoyancy module force [N]
g	Acceleration of gravity [ms^−2^]
$K_{1}$	Variable buoyancy module linear model steady-state gain [ms^−1^V^−1^]
$K_{2}$	Vertical motion linear model steady-state gain [ms^−1^m^−3^]
$K_{22}$	Estimate of the vertical motion linear model steady-state gain [ms^−1^m^−3^]
$K_{O}$	Disturbance observer proportional gain [Nm^−1^]
$K_{OD}$	Disturbance observer derivative gain [Nsm^−1^]
$k_{z}/k_{u}$	Parameter relating the depth and the equivalent depth voltage [Vm^−1^]
*p*	Water pressure [Pa]
$R_{1},R_{2},R_{3}$	Force ramp slopes [Ns^−1^]
$T_{1}$	Variable buoyancy module linear model time constant [s]
$T_{2}$	Vertical motion linear model time constant [s]
$T_{22}$	Estimative of vertical motion linear model time constant [s]
U	Control action [V]
$U_{switch}$	Proposed variable structure controller control action [V]
$U_{Z}$	Equivalent depth voltage [V]
$Vol_{0}$	Ambient pressure volume of the vehicle hull [m^3^]
$Vol_{p}$	Volume of the vehicle hull at pressure *p* [m^3^]
$V_{VBM}$	Variable buoyancy module volume [m^3^]
Z	Vehicle depth [m]
$\hat{Z}$	Disturbance observer vehicle depth estimative [m]
α, β, γ, δ, Ω	Proposed variable structure controller decision regions
$\xi_{K}$	Error of the estimative of the vertical motion linear model steady-state gain [ms^−1^m^−3^]
$\xi_{T}$	Error of the estimative of the vertical motion linear model time constant [s]
ψ	Hull loss of volume per meter depth [m^3^m^−1^]
ρ	Water volumetric mass [kgm^−3^]

## References

[1] Whitt C., Pearlman J., Polagye B., Caimi F., Muller-Karger F., Copping A., Spence H., Madhusudhana S., Kirkwood W., Grosjean L. (2020). Future Vision for Autonomous Ocean Observations. Front. Mar. Sci..

[2] Falcão Carneiro J., Bravo Pinto J., Gomes Almeida F., Cruz N. (2024). Depth control of an underwater sensor platform: Comparison between variable buoyancy and propeller actuated devices. Sensors.

[3] Cao J., Lin R., Liu C., Feng H., Yu C., Yao B., Lian L. (2023). Energy optimal depth control for multimodal underwater vehicles with a high accuracy buoyancy actuated system. Ocean Eng..

[4] Ricks R., Grimmett D., Wakayama C. Passive acoustic tracking for cueing a multistatic active acoustic tracking system. Proceedings of the 2012 Oceans-Yeosu.

[5] Bae I., Hong J. (2023). Survey on the Developments of Unmanned Marine Vehicles: Intelligence and Cooperation. Sensors.

[6] Merchant N.D., Blondel P., Dakin D.T., Dorocicz J. (2012). Averaging underwater noise levels for environmental assessment of shipping. J. Acoust. Soc. Am..

[7] Diviacco P., Nadali A., Iurcev M., Burca M., Carbajales R., Gangale M., Busato A., Brunetti F., Grio L., Viola A. (2021). Underwater Noise Monitoring with Real-Time and Low-Cost Systems, (The CORMA Experience). J. Mar. Sci. Eng..

[8] Guerra M., Thode A.M., Blackwell S.B., Michael Macrander A. (2011). Quantifying seismic survey reverberation off the Alaskan North Slope. J. Acoust. Soc. Am..

[9] Nowacek D.P., Clark C.W., Mann D., Miller P.J., Rosenbaum H.C., Golden J.S., Jasny M., Kraska J., Southall B.L. (2015). Marine seismic surveys and ocean noise: Time for coordinated and prudent planning. Front. Ecol. Environ..

[10] Marques T., Thomas L., Martin S., Mellinger D., Ward J., Moretti D., Harris D., Tyack P. (2012). Estimating animal population density using passive acoustics. Biol. Rev..

[11] Bolgan M., Amorim M.C.P., Fonseca P.J., Di Iorio L., Parmentier E. (2018). Acoustic Complexity of vocal fish communities: A field and controlled validation. Sci. Rep..

[12] Everest F.A., Young R.W., Johnson M.W. (1948). Acoustical Characteristics of Noise Produced by Snapping Shrimp. J. Acoust. Soc. Am..

[13] Cai W., Zhu J., Zhang M., Yang Y. (2022). A Parallel Classification Model for Marine Mammal Sounds Based on Multi-Dimensional Feature Extraction and Data Augmentation. Sensors.

[14] Cauchy P., Heywood K.J., Merchant N.D., Queste B.Y., Testor P. (2018). Wind Speed Measured from Underwater Gliders Using Passive Acoustics. J. Atmos. Ocean. Technol..

[15] Dziak R.P., Lee W.S., Haxel J.H.H., Matsumoto H., Tepp G., Lau T.-K., Roche L., Yun S., Lee C.-K., Lee J. (2019). Hydroacoustic, Meteorologic and Seismic Observations of the 2016 Nansen Ice Shelf Calving Event and Iceberg Formation. Front. Earth Sci..

[16] Hockley C., Butka B. (2019). Can a Conventional Propulsion System Match the Efficiency of an Underwater Glider Buoyancy Engine?. Mar. Technol. Soc. J..

[17] Falcão Carneiro J., Bravo Pinto J., Gomes de Almeida F., Cruz N. (2020). Variable Buoyancy or Propeller-Based Systems for Hovering Capable Vehicles: An Energetic Comparison. IEEE J. Ocean. Eng..

[18] Xie X., Wang Y., Song Y., Yang S., Luo C., Ma W., Lian Y. (2021). Development, optimization, and evaluation of a hybrid passive buoyancy compensation system for underwater gliders. Ocean Eng..

[19] Tran H.N., Nhut Pham T.N., Choi S.H. (2021). Robust depth control of a hybrid autonomous underwater vehicle with propeller torque’s effect and model uncertainty. Ocean Eng..

[20] Xu H., Zhang G.-c., Sun Y.-s., Pang S. (2020). Energy-saving control of long-range autonomous underwater vehicle vertical plane based on human simulating intelligent control method. Int. J. Adv. Robot. Syst..

[21] Zhang L., Pang Y.-J., Su Y.-M., Zhao F.-L., Qin Z.-B. (2008). Expert S-surface control for autonomous underwater vehicles. J. Mar. Sci. Appl..

[22] Carneiro J.F., Pinto J.B., Almeida F.G.d., Cruz N.A. (2023). Model Identification and Control of a Buoyancy Change Device. Actuators.

[23] Falcão Carneiro J., Bravo Pinto J., Gomes de Almeida F., Cruz N.A. (2023). Electrohydraulic and electromechanical buoyancy change device unified vertical motion model. Actuators.

[24] Cauchy P., Heywood K.J., Merchant N.D., Risch D., Queste B.Y., Testor P. (2023). Gliders for passive acoustic monitoring of the oceanic environment. Front. Remote Sens..

